# Manipulating the sequence of food ingestion improves glycemic control in type 2 diabetic patients under free-living conditions

**DOI:** 10.1038/nutd.2016.33

**Published:** 2016-08-22

**Authors:** D Tricò, E Filice, S Trifirò, A Natali

**Affiliations:** 1 Department of Clinical and Experimental Medicine, University of Pisa, Pisa, Italy

## Abstract

Lipid and protein ingested before carbohydrate reduce postprandial hyperglycemia. We tested feasibility, safety and clinical efficacy of manipulating the sequence of nutrient ingestion in patients with type 2 diabetes (T2D). After a 4-week run-in, 17 T2D patients were randomized to either a control diet (CD) or to an experimental diet (ED) allowing the consumption of high-carbohydrate foods only after high-protein and high-fat foods at each main meal (lunch+dinner). Both diets were accurately followed and neutral on arterial blood pressure, plasma lipids and indices of hepatic and kidney function. After 8 weeks, in spite of a similar reduction of body weight (ED −1.9 95% confidence interval (−3.4/−0.4)kg, *P*<0.03; CD −2.0 (−3.6/−0.5)kg, *P*<0.02) and waist circumference (ED −2.9 (−4.3/−1.5)cm, *P*<0.002; CD −3.3 (−5.9/−0.7)cm, *P*<0.02), the ED only was associated with significant reductions of HbA1c (−0.3 (−0.50/−0.02)%, *P*<0.04), fasting plasma glucose (−1.0 (−1.8/−0.3)mmol l^−1^, *P*<0.01), postprandial glucose excursions (lunch −1.8 (−3.2/−0.4)mmol l^−1^, *P*<0.01; dinner: −1.0 (−1.9/−0.1)mmol l^−1^, *P*<0.04) and other indices of glucose variability (s.d.: −0.5 (−0.7/−0.2)mmol l^−1^, *P*<0.02; Coefficient of variation: −6.6 (−10.4/−2.7)%, *P*<0.02). When compared with the CD, the ED was associated with lower post-lunch glucose excursions (*P*<0.02) and lower glucose coefficients of variation (*P*<0.05). Manipulating the sequence of nutrient ingestion might reveal a rapid, feasible, economic and safe strategy for optimizing glucose control in T2D.

## Introduction

Lipid and protein ingested before carbohydrate, as a ‘preload', have been shown to acutely improve glucose tolerance, mainly by delaying gastric emptying and by enhancing insulin secretion.^1, 2, 3, 4, 5, 6^ Indeed, we recently reported that a small mixed non-glucidic preload markedly improved glucose tolerance by delaying glucose absorption, enhancing beta cell function and reducing insulin clearance in patients with type 2 diabetes.^1,2^ Whether these acute effects persist over time is unclear.^7,8^ Furthermore, adding a nutrient preload to each meal to improve postprandial glucose control could be unfeasible and/or increase the total daily caloric intake. As recently suggested by an acute pilot study,^9^ we tested the hypothesis that manipulating the sequence of food consumption during each main meal (i.e., high-protein and high-lipid foods before carbohydrate) would exploit the same marked hypoglycemic effects of non-glucidic nutrient preloads, revealing a simple, safe and effective strategy to improve glucose control in type 2 diabetic patients.

## Subjects and methods

### Study population

Twenty well-controlled type 2 diabetic patients were enrolled. The inclusion criteria were age 50–75 years, body mass index (BMI) 26–35 kg m^−2^, stable weight for at least 6 months, glycated hemoglobin 48–58 mmol mol^−1^, disease duration ⩽5 years. None had diseases other than diabetes or was taking medications other than metformin and/or sitagliptin that could potentially interfere with carbohydrate absorption and/or metabolism. The institutional Ethics Committee approved the study and all participants provided written informed consent before inclusion in the study.

### Study design

This was a parallel, randomized, open clinical trial. Participants were evaluated on four consecutive visits separated by 28±2 days at 08:00 am after an overnight fast. On each occasion, body weight, fat mass (FM) and basal metabolic rate (BMR) were assessed by bioelectrical impedance (TBF-300 Body Composition Analyzer, Tanita Corporation, Arlington Heights, IL, USA); waist and hip circumferences, and systolic and diastolic blood pressure were measured according to standard procedures. Blood samples were collected at study entry (visit 1), after 28 days of run-in (visit 2) and after 56 days of diet (visit 4) for the measurement of blood glucose, glycated hemoglobin, total cholesterol, LDL cholesterol, HDL cholesterol, triglycerides, and standard indices of renal, hepatic, pancreatic and thyroid function. Volunteers were also asked to measure their blood glucose concentrations by glucometer (Contour XT, Bayer HealthCare LLC, Whippany, NJ, USA) once a week six times in a single day (before and two hours after breakfast, lunch, and dinner) for the full length of the study. The total daily caloric need was estimated in each volunteer by adding the BMR to the individual caloric expenditure during working and leisure time physical activity. On visit 2, volunteers were randomized into two different groups. Subjects from control group were asked to follow an 8-week standard balanced mild-hypocaloric diet (control diet (CD)).^10^ Each subject received a dietary plan with the food composition of three typical meals (breakfast, lunch and dinner) and a table of possible substitutions with variable equicaloric amounts of different foods. Meals and variants were pondered to yield a caloric deficit of ~200 kcal per day with respect to the total daily caloric need, to produce an expected weight loss of ~1 kilogram a month. Patients from the experimental group received the same diet plan in terms of food quality and quantity (experimental diet, ED). In addition, they received indications on macronutrient composition of foods and were strongly recommended to fix the sequence of macronutrient ingestion at each main meal (lunch and dinner), so as to eat high-carbohydrate-containing foods (e.g., bread, pasta, potatoes) preferably after the ingestion of high-protein and high-fat foods (e.g., meat, cheese, fish). A typical main meal in the ED was so composed of meat as the first course, then vegetables, bread and/or pasta and fruit. All volunteers were asked to report their overall compliance to the caloric content and to the sequence of nutrients of the prescribed diet by checking on an *ad hoc* designed form at each meal.

### Statistical analysis

Data are shown as mean±s.e.m. The changes induced by our experimental maneuver were evaluated by using either Wilcoxon signed rank or Mixed-model multivariate analysis of variance (MANOVA) for repeated measures with time as within-subject factor, diet as between subjects factor, and the interaction time*diet *as* main outcome variable. Data from self-monitoring of blood glucose were analyzed by calculating mean 2-hours glucose increments over pre-meal values for each meal (breakfast, lunch, dinner) and by calculating mean concentrations, s.d. and percentage coefficients of variation (CV; s.d./mean ratio) of lunch+dinner glucose values over 4 days (on week apart) during the run-in, the first and the second 4 weeks of diet.^11^ Statistical analyses were performed using JMP 9.0 (SAS Institute Inc., Cary, NC, USA). A value of *P*⩽0.05 was considered statistically significant.

## Results

### Characteristics of the study population

Three volunteers were excluded due to their poor compliance to the study protocol, nine were included in the control group (age 64±8 years, 6 males and 3 females, 4 on metformin and 2 on metformin+sitagliptin therapy) and eight in the experimental group (age 65±7 years, 6 males and 2 females, 4 on metformin and 1 on metformin+sitagliptin therapy). Clinical and metabolic characteristics were similar between the two study groups (Table 1). Although a proper subgroup analysis was not performed due to the small number of subjects, neither metformin nor sitagliptin use has reasonably affected study results, since subjects taking medications showed no large difference in glycemic responses compared with other group members. Self-reported dietary compliance at each main meal was >95% in terms of caloric intake and >90% in terms of food sequence. None of the patients randomized to the ED complained of any distress associated with the fixed sequence of nutrient consumption during their two main meals (lunch and dinner).

### Clinical variables

The two diet regimens produced similar (diet and time*diet effects=ns by MANOVA) and close-to-the-expected reductions in body weight (ED −1.9 kg, 95% confidence interval (CI) (−3.4/−0.4); CD −2.0 kg, 95% CI (−3.6/−0.5); time effect *P*<0.003), BMI (ED −0.7 kg m^−2^, 95% CI (−1.2/−0.2); CD −0.7 kg m^−2^, 95% CI (−1.2/−0.2); time effect *P*<0.002), FM (ED −1.5%, 95% CI (−2.8/−0.3); CD –0.8%, 95% CI (−1.8/0.2); time effect *P*<0.02) and waist circumference (ED −2.9 cm, 95% CI (−4.3/−1.5); CD −3.3 cm, 95% CI (−5.9/−0.7); time effect *P*<0.002) (Table 1). No differences were found in serum lipids or systolic and diastolic blood pressure values in any of the four visits in the two study groups (Table 1). Neither diet affected renal, hepatic, pancreatic and thyroid function indices (data not shown).

### Glucose control variables

**Figure 1:**
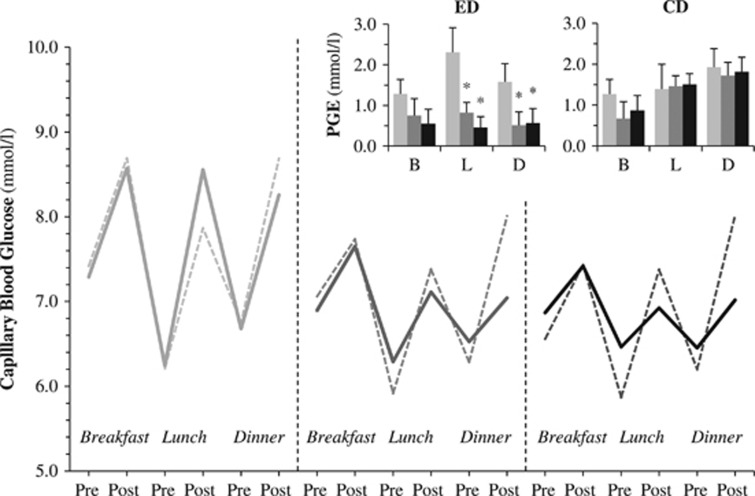
Mean capillary blood glucose concentrations and postprandial glucose excursions (PGE) (top right corner) before and after breakfast (B), lunch (L) and dinner (D) during the run-in (light gray), the first 4 weeks (dark gray) and the second 4 weeks (black) of experimental diet (ED, continuous line) and control diet (CD, dashed line). *P<0.05 by Wilcoxon in comparison with the run-in PGE value.

After 8 weeks, the ED produced an improvement of the overall glucose control, as assessed by the reduction of glycated hemoglobin (−0.3%, 95% CI (−0.50/−0.02), *P*<0.04 by Wilcoxon) (Table 1). This was associated to a decline of 1.0 mmol l^−1^ (95% CI (−1.8/−0.3), *P*<0.01) in fasting plasma glucose and of 0.8 mmol l^−1^ (95% CI (−1.4/−0.2), *P*<0.04) in mean lunch+dinner glucose (Table 1), and to a marked reduction of postprandial glucose excursions (lunch: −1.8 mmol l^−1^, 95% CI (−3.2/−0.4), *P*<0.01; dinner: −1.0 mmol l^−1^, 95% CI (−1.9/−0.1), *P*<0.04) and other indices of glucose variability (SD −0.5 mmol l^−1^, 95%CI (−0.7/−0.2), *P*<0.02; CV −6.6%, 95% CI (−10.4/−2.7), *P*<0.02) (Figure 1). The CD produced a non-significant reduction of glycated hemoglobin (−0.3%, 95% CI (−0.6/0.1), *P*=0.09) and fasting plasma glucose (−0.7 mmol l^−1^, 95% CI (−1.6/0.2), *P*=0.06), and it failed to improve postprandial glucose excursions and other glucose variability indices (Table 1). Among these variables, the time*diet effect by MANOVA was statistically significant for post-lunch glucose excursions (*P*<0.04) and for the CV of glucose concentrations (*P*<0.05).

## Discussion

This study demonstrates that by only manipulating the sequence of nutrient ingestion it is possible to improve glycemic excursions in type 2 diabetic patients in free-living conditions, and that this intervention is safe and well accepted. More in general, it proves the concept that it is effective and feasible to rely upon the physiologic responses acutely activated by nutrient ingestion (i.e., nutrient sensing^12^) to improve glucose homeostasis. Participants were instructed to consume high-carbohydrate-containing foods only after non-glucidic nutrients, to exploit and combine the well-known positive effects of lipid and protein on glucose tolerance^1,3,4,5,6^ without increasing the total amount of foods and without requiring supplements (artificial formula) that might be expensive and poorly accepted. Despite the high variability inherent to the real-life setting and the small populations, the time course of blood glucose self-monitoring revealed that an overall reduction in glycemic variability, particularly at the manipulated meals (lunch and dinner), was already evident at the first month of diet and sustained through the following 4 weeks (Figure 1). Accordingly, the effects of the ED on glucose variability indices were not related to the extent of individual weight loss. If applied also to the breakfast (scarcely feasible for Italian habits), the overall effect of this dietary intervention on glucose control, namely on glycated hemoglobin, would have probably been greater. Although conceived on the bases of the same experimental evidences, our approach may have several advantages with respect to the already proposed protein supplement preloads.^7^ First, the physiological combination of lipid and protein is likely to be more effective, by acting on multiple targets;^1,13^ indeed, the effect on glucose tolerance of protein alone, though persistent, was quantitatively small.^7^ Second, with our approach the daily caloric intake and the proportions of macronutrients are not altered; as expected, the ED has no impact on body weight nor it alters body mass composition, lipid profile or indices of renal function.

## Conclusions

In conclusion, this pilot study supports the concept that manipulating the sequence of nutrient ingestion might reveal a useful, feasible and inexpensive strategy for long-term management of type 2 diabetes and provides encouragement for further longer-term and larger clinical trial.

## Figures and Tables

**Table 1:** Clinical and metabolic variables

	*Experimental diet*				*Control diet*			
	−*4 weeks*	*0*	*4 weeks*	*8 weeks*	−*4 weeks*	*0*	*4 weeks*	*8 weeks*
Weight (kg)	85.6±2.4	84.9±2.3	83.4±2.5^a^	83.0±2.5^b^	85.3±5.1	84.8±5.1	83.2±4.7^a^	82.7±4.7^b^
BMI (kg m^−2^)	31.1±1.3	30.9±1.3	30.2±1.2^a^	30.2±1.2^b^	30.5±1.2	30.3±1.2	29.8±1.1^a^	29.6±1.2^b^
Fat mass (%)	32.2±3.3	31.3±2.8	29.9±2.8	29.8±3.0^b^	31.0±2.8	30.9±2.7	30.2±2.6	30.1±2.6
Fat-free mass (%)	60.9±1.3	61.0±1.1	61.2±1.0	61.4±1.2	58.9±4.2	58.5±4.3	58.0±3.9	57.8±3.9
Waist (cm)	104±2	103±2	102±2	100±2^b^^,^^c^	105±4	104±4	104±4	101±4^b^^,^^c^
Waist/hip ratio	0.99±0.02	0.99±0.01	0.98±0.02	0.97±0.02	1.00±0.02	0.99±0.02	1.00±0.03	0.99±0.02
Systolic blood pressure (mm Hg)	134±4	136±9	125±6	131±7	129±5	127±4	133±4	128±3
Diastolic blood pressure (mm Hg)	86±3	76±7	81±3	82±4	84±4	83±4	79±2	80±1
HbA_1c_ (%)	6.7±0.2	6.7±0.2	—	6.4±0.2^b^	6.8±0.1	6.8±0.1	—	6.6±0.1
HbA_1c_ (mmol mol^−1^)	49.3±1.7	49.4±2.0	—	46.7±1.7^b^	51.3±1.6	51.2±1.6	—	48.4±1.4
Fasting plasma glucose (mmol l^−1^)	6.9±0.4	7.1±0.5	—	6.1±0.3^b^	6.8±0.3	6.4±0.3	—	5.6±0.4
PGE breakfast (mmol l^−1^)	—	1.3±0.4	0.8±0.3	0.5±0.3	—	1.2±0.4	0.7±0.3	0.9±0.5
PGE lunch (mmol l^−1^)	—	2.3±0.6	0.8±0.3^a^	0.5±0.3^b^	—	1.3±0.4	1.5±0.5	1.5±0.5
PGE dinner (mmol l^−1^)	—	1.5±0.5	0.5±0.3^a^	0.6±0.4^b^	—	1.7±0.6	1.6±0.4	1.8±0.6
Mean glucose (mmol l^−1^)	—	7.5±0.4	6.7±0.4^a^	6.7±0.3^b^	—	7.4±0.3	6.9±0.3	6.9±0.3
s.d. (mmol l^−1^)	—	1.5±0.2	1.1±0.2	0.9±0.1^b^	—	1.7±0.2	1.4±0.2	1.5±0.2
Coefficient of variation (%)	—	19.6±2.2	16.6±1.9	13.0±1.2^b^	—	22.9±2.1	19.3±2.2	21.3±2.2

Abbreviations: PGE, postprandial glucose excursions.

a 4 weeks vs 0, *P*<0.05;

b 8 weeks vs 0, *P*<0.05;

c 8 weeks vs 4 weeks, *P*<0.05. PGE are the mean 2-hours glucose increments over pre-meal values following each meal during the run-in (−4 to 0 weeks), the first (0–4 weeks) and the second (4–8 weeks) 4 weeks of diet. Data are mean±s.e.m.
